# Gastrodin Reduces Blood Pressure by Intervening with RAAS and PPAR*γ* in SHRs

**DOI:** 10.1155/2015/828427

**Published:** 2015-10-26

**Authors:** Wei Liu, Lingyan Wang, Jiahui Yu, Patrick Fordjour Asare, Ying-Qiang Zhao

**Affiliations:** ^1^Institute of Traditional Chinese Medicine, Tianjin University of Traditional Chinese Medicine, Tianjin, China; ^2^Department of Cardiology, Second Affiliated Hospital of Tianjin University of Traditional Chinese Medicine, Tianjin, China

## Abstract

Gastrodin is a bioactive compound extracted from traditional Chinese medicine, *Gastrodia elata*  Bl. It has a definite effect on reducing blood pressure in hypertensive patients. However, the mechanisms of gastrodin in lowering blood pressure still remain unclear. In this study, 4 weeks of administration of gastrodin (100 mg/kg/d intraperitoneally injected) decreased the systolic blood pressure (SBP) in spontaneously hypertensive rats (SHRs) (190.2 ± 8.9 versus 169.8 ± 6.4, *P* < 0.01). Among SHRs receiving gastrodin treatment, angiotensin II (Ang II) and aldosterone (ALD) in serum were significantly decreased (2022.1 ± 53.0 versus 1528.7 ± 93.9, 213.33 ± 35.17 versus 179.65 ± 20.31, and *P* < 0.01, *P* < 0.05, resp.) and dramatically downregulated expression of angiotensin type 1 receptor (AT1R) (4.9 ± 0.9 versus 2.6 ± 0.9, *P* < 0.05) in myocardium in both mRNA and protein levels compared with their corresponding groups without gastrodin treatment. Additionally, gastrodin increased the mRNA expression (0.18 ± 0.07 versus 0.82 ± 0.10, *P* < 0.01) and protein synthesis (0.40 ± 0.10 versus 0.34 ± 0.10, *P* < 0.01) of peroxisome proliferator-activated receptor *γ* (PPAR*γ*) in myocardium tissues. Overall, our data demonstrated that gastrodin was able to decrease the SBP in SHR. Furthermore, this study showed that gastrodin intervened with the renin-angiotensin-aldosterone system (RAAS) and PPAR*γ* effectively, which indicates its antihypertensive mechanism.

## 1. Introduction

As one of the most common chronic diseases in the world, hypertension accelerates the progression of cardiovascular disease and severely threatens human health. It has been shown that the renin-angiotensin-aldosterone system (RAAS) plays a major role in the initiation and progression of hypertension [1]. RAAS is a cascade with effector molecules such as angiotensin II (Ang II) and aldosterone (ALD). Ang II has a strong biological activity in constricting blood vessels, enhancing aldosterone secretion, and ultimately elevating blood pressure level. Aldosterone enhances the reabsorption of sodium and water, thus increasing blood volume and blood pressure [2]. Therefore, inhibiting the activation of RAAS is one of the main strategies to lower blood pressure in patients with hypertension.

Peroxisome proliferator-activated receptor (PPAR) *γ* is a nuclear transcription factor regulated by ligands which are expressed in the cardiovascular system [3]. It has been reported that PPAR*γ* can regulate the gene expression related with RAAS and may play a regulatory role in blood pressure modulation [4].

**Figure 1:**
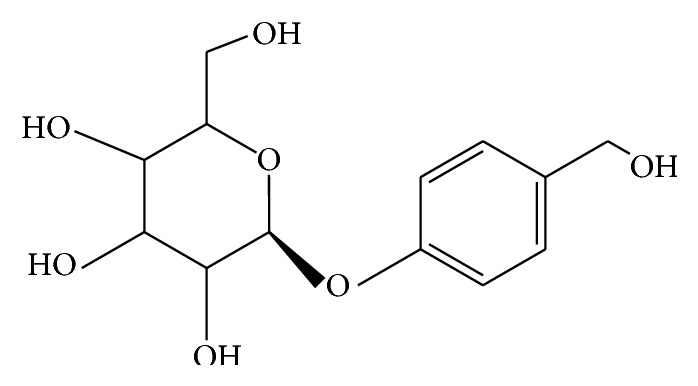
Structure of gastrodin.

Gastrodin (PubChemCID:115067(2R,3S,4S,5R,6S)-2-(hydroxymethyl)-6-[4-(hydroxymethyl)phenoxy]oxane- 3,4,5-triol, Figure 1) is one of the major bioactive components extracted from the Chinese herb* Gastrodia elata* Bl. (Figure 1). Gastrodin injection has been extensively used to treat cardiovascular and cerebrovascular diseases in China and has a certain efficacy to lower blood pressure in hypertensive patients [5]. However, the related mechanisms still remain cryptic. Therefore, we aimed to determine whether gastrodin could attenuate blood pressure by regulating RAAS and PPAR*γ*. It is important to note that gastrodin is used clinically to manage cardiovascular disease and that the heart is the main target organ in the management of cardiovascular diseases. In this regard, we used echocardiography to evaluate cardiac function after gastrodin treatment.

Clinical and experimental evidence suggests that cardiac renin-angiotensin system (RAS) plays an important role in the regulation of SBP. Of note, PPAR*γ* expression occurs in the heart and has been shown to possess key regulatory function on the cardiac RAS expression level. Our explicit objective was to study the biological action of gastrodin and its therapeutic significance on the regulation of PPAR*γ*, ACE, and AT1R in the heart. We found that activation of cardiac PPAR*γ* correlated with reduced expression level of myocardial ACE and AT1R expression levels. The subsequent reduction in blood pressure level suggests that gastrodin could reduce blood pressure by elevating the expression of PPAR*γ* which negatively regulates cardiac ACE and AT1R.

## 2. Materials and Methods

### 2.1. Animals and Treatment

Ten-week-old male Wistar-Kyoto (WKY) and spontaneously hypertensive rats (SHRs) were obtained commercially from Vital River Laboratories, Beijing, China. SHRs were randomly divided into model (SHR) and treatment groups (GAS) with 8 rats in each group. Eight WKY belonged to the control group (WKY). Rats were housed in a controlled environment (22 ± 2°C and 50%  ±  5% humidity) receiving a circadian rhythm of 12 h/12 h light/dark. Rats were allowed food and water* ad libitum*. Bedding was refreshed daily for every cage. The first two weeks was the adaptation period, and rats were not given any intervention. Gastrodin therapy started from the third week during which rats were under therapeutic treatment. SHRs in the treatment group were injected intraperitoneally with gastrodin at the dose of 100 mg/(kg·d) for 4 weeks. The dose of gastrodin administration was arrived at by critical assessment of clinical dosage and analysis of previous studies [6]. Body weight and blood pressure of the rats were measured every week. All the procedures were approved by the Animal Ethics Review Committee of Tianjin University of Traditional Chinese Medicine.

### 2.2. Reagents

Gastrodin injections were purchased from Hainan Helpson Medicine & Biotechnique Co., Ltd. The concentration of gastrodin was 100 mg/mL, and it was stable for 2 years at room temperature. ELISA kit of aldosterone and angiotensin II were purchased from Beijing Sino-UK Institute of Biological Technology. Western blot reagents were purchased from Sigma, while antibodies were purchased from Santa Cruz. Real-time quantitative PCR reagents were purchased from Tianjin Hao Yang Biological Manufacture Co., Ltd., and the primers were synthesized by Sangon Biotech (Shanghai) Co., Ltd.

### 2.3. Monitoring of Blood Pressure, Heart Rate, and Body Weight

Blood pressure (systolic blood pressure), heart rate, and body weight of rats were monitored once a week. Systolic blood pressure (SBP) and heart rate were monitored by the noninvasive tail-cuff method using animal sphygmomanometer (BP98AWU, Softron, Japan). All the blood pressure and heart rate measurements were performed on conscious animals. For each SHR and WKY rat, blood pressure and heart rate were measured with multiple readings, until 15 stable measurements in a row were obtained. Data were calculated as an average of blood pressure and heart rate values.

### 2.4. Cardiac Function Study by Echocardiography

Echocardiography was done by an observer blinded to the experiment, and measurements were taken before sacrifice. Echocardiography was performed by Visual Sonics Vevo 2100 imaging system. The LV was assessed in both parasternal long-axis and short-axis views at a frame rate of 50 Hz. End-systole or end-diastole was defined as the phase in which the smallest or largest area of LV, respectively, was obtained. Left ventricular end-diastolic diameter (LVEDD) and left ventricular end-systolic diameter (LVESD) were measured from the LV M-mode tracing with a sweep speed of 50 mm/s at the mid-papillary muscle level. These parameters were used to determine left ventricular ejection fraction.

### 2.5. Serum Collection and Harvest of the Tissue

After abdominal anesthesia in rats with chloral hydrate (5%, 6 mL/kg, i.p.), blood samples were collected via abdominal aorta puncture. Serum was then prepared by centrifugation of the collected blood (2000 rpm for 20 min). Serum samples were stored at −80°C and used to determine the levels of aldosterone (ALD) and angiotensin II (Ang II) with ELISA kits in a blinded manner following the manufacturer's instructions. Heart was removed from each rat.

### 2.6. Quantitative Real-Time Reverse Transcription Polymerase Chain Reaction (qRT-PCR) Analysis

Total RNA was isolated from the myocardium tissues (*n* = 8 per group) using TRIZOL reagent (Tianjin Hao Yang Biological manufacture Co., Ltd., China). RNA was reverse-transcribed using SuperScript First Strand cDNA System (Invitrogen, Carlsbad, CA) according to the manufacturer's instructions.

The following primer sequences were used: *β*-actin (NM_031144), forward (TCA GGT CAT CAC TAT CGG CAA), reverse (AGC ACT GTG TTG GCA TAG AGG), ACE (NM_012544.1), forward (AAC AGG TTC GTG GAG GAG TAT), reverse (CAG GTG CCA TAT TTC AAG GTA). AT1R (NM_030985.4), forward (ATC TCG CCT TGG CTG ACT TAT), reverse (GAA GGA ACA CAC TGG CGT AGA), and PPAR*γ* (NM_001145366.1), forward (AAG GGT GCC AGT TTC GAT CC), reverse (TAT TCA TCA GGG AGG CCA GCA). The sizes of the PCR products amplified with the primers were *β*-actin, 169 bp, ACE, 161 bp, AT1R, 150 bp, and PPAR*γ*, 159 bp, respectively. In preliminary experiments, we confirmed that the efficiency of these primer pairs is comparable (data not shown). qRT-PCR was done using SYBR Green PCR master mix (Applied Biosystems) in a total volume of 20 *μ*L on the 7900HT fast real-time PCR system (Applied Biosystems) as follows: 95°C for 15 min, 40 cycles of 95°C for 20 s, and 57°C for 20 s. A dissociation procedure was performed to generate a melting curve for confirmation of amplification specificity. *β*-actin was used as the reference gene. The relative levels of gene expression were represented as ΔCt = Ct_gene_ − Ct_reference_, and the fold change of gene expression was calculated by the 2^−ΔΔCt^ method [7].

### 2.7. Western Blotting Assay

Western blotting assay was performed to determine protein expression of AT1R and PPAR*γ* (*n* = 4 per group). The myocardium tissue was homogenized in the lysis buffer using an ultrasound homogenizer at 50 Hz. The lysate was then centrifuged. The protein concentration of the supernatant was measured with the Bradford protein assay. Proteins were loaded into 8% SDS-polyacrylamide gels and transferred to PVDF membranes. After blocking in nonfat milk, the membranes were exposed to a rabbit polyclonal antibody against AT1R or PPAR*γ* or *β*-actin overnight at 4°C. After incubation with HRP-linked secondary antibodies, the immune complexes were visualized with chemiluminescence (ECL Blotting Analysis System; Amersham, Arlington Heights, IL) and exposed to X-ray film, measured with Image J software, and normalized to *β*-actin.

### 2.8. Statistical Analysis

All results were expressed as mean ± S.E.M. Statistical comparisons between different groups were performed by one-way ANOVA with Dunnett's multiple comparison posttest. Differences with *P* value less than 0.05 were considered statistically significant.

## 3. Results

### 3.1. General Health of Rats in Each Group

**Figure 2:**
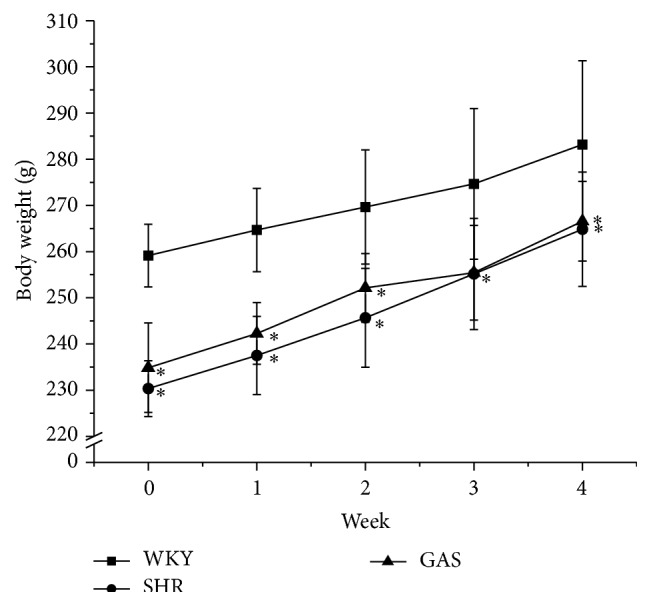
Effects of 4-week treatment with gastrodin on body weight of rats. Ten-week-old male Wistar-Kyoto (WKY) and spontaneously hypertensive rats (SHRs) were obtained. SHRs were randomly divided into model (SHR) and treatment groups (GAS). WKY belonged to control group (WKY). The first two weeks was adaptation period. Starting from the third week, rats in GAS group were intraperitoneally injected with gastrodin at the dose of 100 mg/(kg·d) for 4 weeks. The rest of the rats were not given any intervention other than providing food and water. The body weights were recorded weekly during the experimental period. Values are shown as means ± SEM. ^*∗*^ *P* < 0.05 versus WKY.

**Figure 3:**
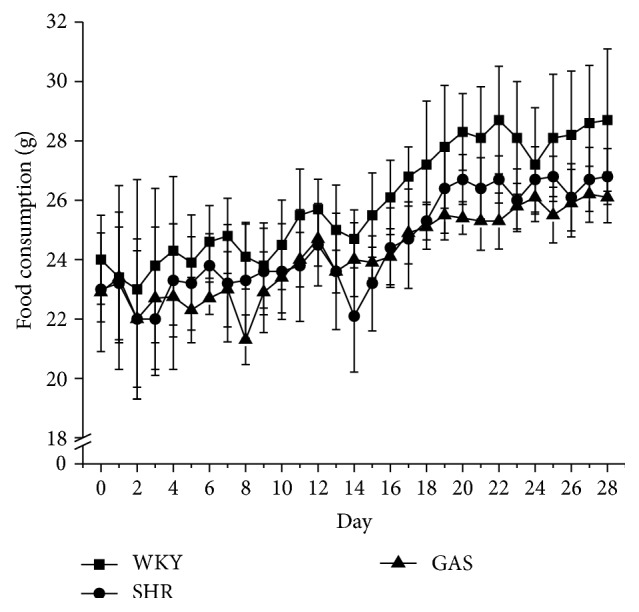
Effects of 4-week treatment with gastrodin on food consumption of rats. Ten-week-old male Wistar-Kyoto (WKY) and spontaneously hypertensive rats (SHRs) were obtained. SHRs were randomly divided into model (SHR) and treatment groups (GAS). WKY belonged to control group (WKY). The first two weeks was adaptation period. Starting from the third week, rats in GAS group were intraperitoneally injected with gastrodin at the dose of 100 mg/(kg·d) for 4 weeks. The rest of the rats were not given any intervention. The food consumption was recorded daily during the experimental period. Values are shown as means ± SEM.

**Figure 4:**
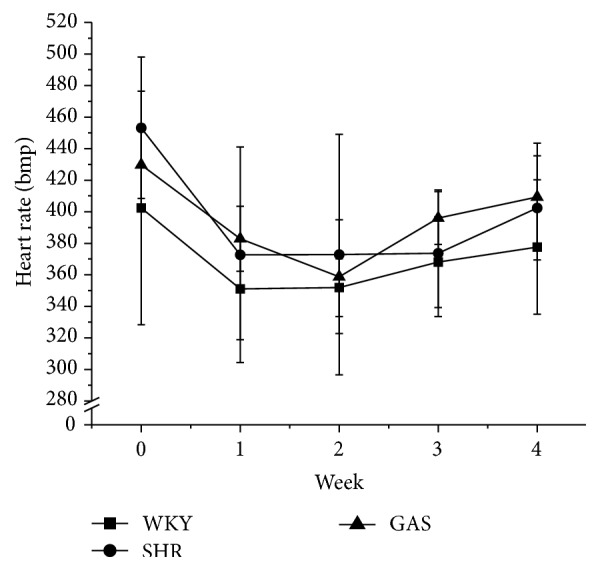
Effects of 4-week treatment with gastrodin on heart rate of rats. Ten-week-old male Wistar-Kyoto (WKY) and spontaneously hypertensive rats (SHRs) were obtained. SHRs were randomly divided into model (SHR) and treatment groups (GAS). WKY belonged to control group (WKY). The first two weeks was adaptation period. Starting from the third week, rats in GAS group were intraperitoneally injected with gastrodin at the dose of 100 mg/(kg·d) for 4 weeks. The rest of the rats were not given any intervention. The heart rate was recorded weekly during the experimental period. Values are shown as means ± SEM.

Gastrodin did not significantly affect the body weight of the rats in this study; however, all rat weights increased during the experimental period. WKY's weights were more than the other two groups from the beginning to the end of the study. All the differences were statistically significant (Figure 2). Daily intraperitoneal injection of gastrodin did not affect food consumption (Figure 3). Moreover, gastrodin did not affect heart rate (Figure 4).

### 3.2. Systolic Blood Pressure

**Figure 5:**
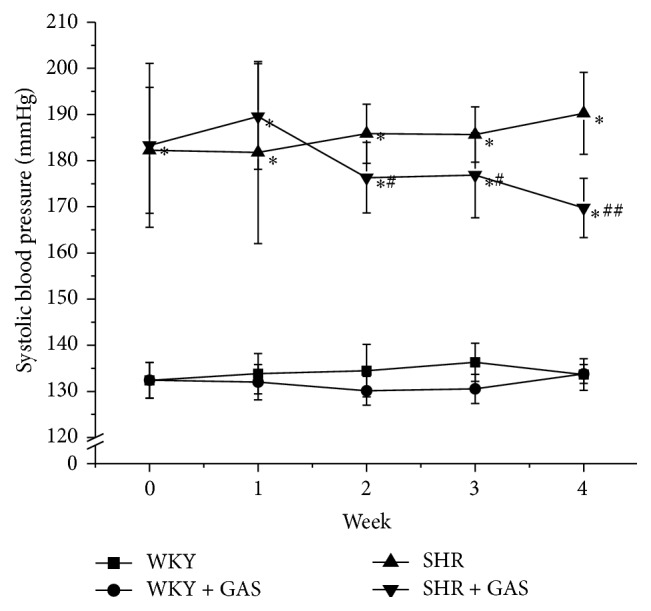
Effects of 4-week treatment with gastrodin on systolic blood pressure (SBP) of rats. Ten-week-old male Wistar-Kyoto (WKY) and spontaneously hypertensive rats (SHRs) were obtained. SHRs were randomly divided into model (SHR) and treatment groups (SHR + GAS). WKY were randomly divided into control (WKY) and treatment groups (WKY + GAS). The first two weeks was adaptation period. Starting from the third week, rats in SHR + GAS and WKY + GAS groups were intraperitoneally injected with gastrodin at the dose of 100 mg/(kg·d) for 4 weeks. The rest of the rats were not given any intervention. The SBP of the animals was recorded weekly during the experimental period. Values are shown as means ± SEM. ^*∗*^ *P* < 0.01 versus WKY; ^#^ *P* < 0.05, ^##^ *P* < 0.01 versus SHR.

At the baseline, the systolic blood pressure (SBP) was no different than SHR and GAS. After two weeks of treatment, SBP of GAS began to reduce and was significantly decreased compared with SHR (*P* < 0.05). There was also a recorded steady reduction of SBP in GAS treated group, while elevated level of SBP in SHR was observed. Throughout the experiment, SBP of WKY was lower than in SHR and GAS (*P* < 0.01) (Figure 5). The data about the effects of gastrodin in the normotensive rats are not shown. First of all, there was no significant difference in the indices on the normotensive rats after daily administration of gastrodin injection.

### 3.3. Cardiac Function

After four weeks of treatment, there was no significant difference in cardiac function among the three groups (Table 1).

### 3.4. Effects of Gastrodin on RAAS

**Figure 6:**
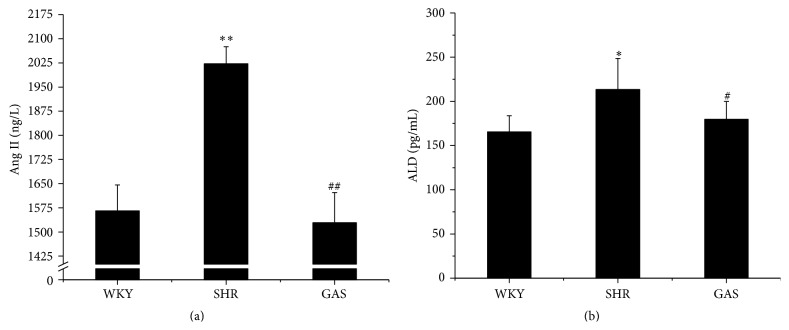
Effects of 4-week treatment with gastrodin on Ang II and ALD in serum of rats. (a) After 4-week treatment with gastrodin, levels of Ang II in serum were determined with ELISA kits. (b) After 4-week treatment with gastrodin, levels of ALD in serum were determined with ELISA kit. Values are shown as means ± SEM. ^*∗*^ *P* < 0.05, ^*∗∗*^ *P* < 0.01 versus WKY; ^#^ *P* < 0.05, ^##^ *P* < 0.01 versus SHR.

Since gastrodin showed effect on SBP in SHR, we explored the possible mechanisms. Previous studies have shed more light on the role RAAS plays in regulating blood pressure levels. In this regard, we sought to determine the effects of gastrodin on RAAS* in vivo*. After four weeks of treatment, the serum level of Ang II in GAS was lower than in SHR (*P* < 0.01). Surprisingly, the Ang II serum level in GAS was also lower than in WKY (Figure 6(a)). Gastrodin also significantly reduced ALD level in serum compared with SHR (*P* < 0.05). There was no significant difference between WKY and GAS treatment groups in the serum level of ALD (Figure 6(b)).

**Figure 7:**
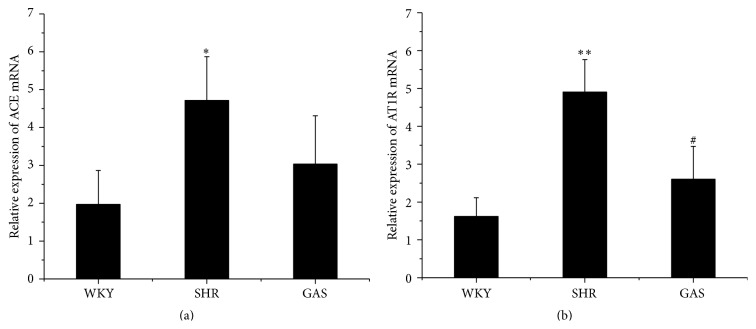
Effects of 4-week treatment with gastrodin on mRNA expression of ACE and AT1R. (a) After 4-week treatment with gastrodin, mRNA expressions of ACE in myocardium tissues were determined by qRT-PCR analysis. (b) After 4-week treatment with gastrodin, mRNA expressions of AT1R in myocardium tissues were determined by qRT-PCR analysis. Values are shown as means ± SEM. ^*∗*^ *P* < 0.05, ^*∗∗*^ *P* < 0.01 versus WKY; ^#^ *P* < 0.05, ^##^ *P* < 0.01 versus SHR.

**Figure 8:**
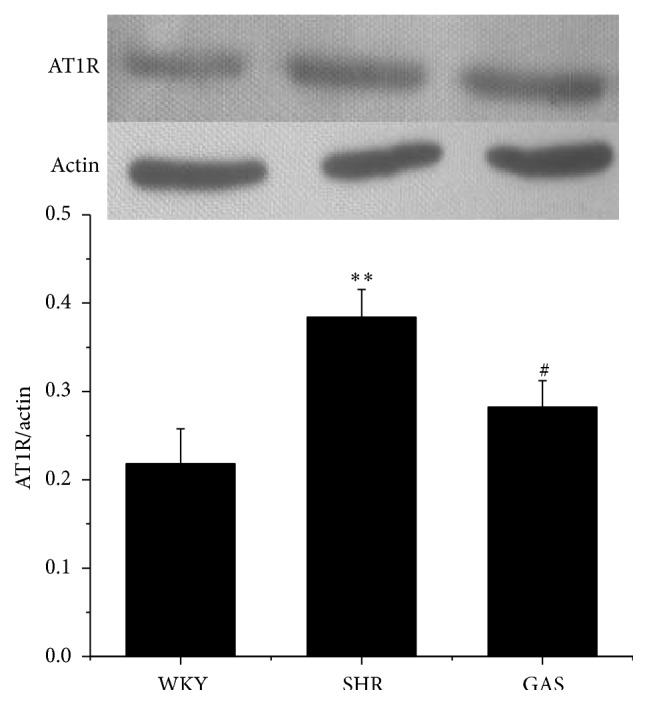
Effects of 4-week treatment with gastrodin on protein levels of AT1R in myocardium tissues of rats. After 4-week treatment with gastrodin, protein levels of AT1R in myocardium tissues were determined by western blotting assay. Values are shown as means ± SEM. ^*∗*^ *P* < 0.05, ^*∗∗*^ *P* < 0.01 versus WKY; ^#^ *P* < 0.05, ^##^ *P* < 0.01 versus SHR.

We further examined the mRNA expression of ACE and AT1R by qRT-PCR. After four weeks of treatment, compared with SHR, the mRNA expression of ACE in GAS declined, but the difference was not statistically significant (Figure 7(a)). Meanwhile, the mRNA expression of AT1R was lower in GAS than in SHR, and the difference was statistically significant (*P* < 0.05) (Figure 7(b)). There was no significant difference in mRNA expression levels of ACE and AT1R between WKY and GAS groups (Figures 7(a) and 7(b)). Next, we examined the protein level of AT1R in myocardium by western blotting. The protein level of AT1R in GAS was significantly lower than in SHR (*P* < 0.05), while it was relatively higher compared with WKY (Figure 8).

### 3.5. Effects of Gastrodin on PPAR*γ*

Considering that PPAR*γ* had relevance with RAAS, we further examined the mRNA expression and protein level of PPAR*γ* in myocardium.

**Figure 9:**
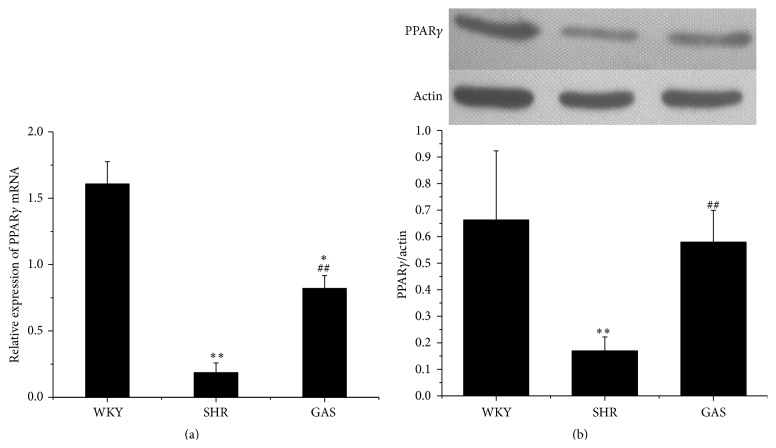
Effects of 4-week treatment with gastrodin on PPAR*γ* in myocardium tissues of rats. (a) After 4-week treatment with gastrodin, mRNA expressions of PPAR*γ* in myocardium tissues were determined by qRT-PCR analysis. (b) After 4-week treatment with gastrodin, protein levels of PPAR*γ* in myocardium tissues were determined by western blotting assay. Values are shown as means ± SEM. ^*∗*^ *P* < 0.05, ^*∗∗*^ *P* < 0.01 versus WKY; ^#^ *P* < 0.05, ^##^ *P* < 0.01 versus SHR.

After four weeks of treatment, the mRNA expression of PPAR*γ* in GAS was higher than in SHR (*P* < 0.01). And it was lower than in WKY (*P* < 0.05) (Figure 9(a)).

Through western blotting, the protein level of PPAR*γ* in GAS was significantly higher than in SHR (*P* < 0.01), while it was relatively lower compared with WKY (Figure 9(b)).

## 4. Discussion

Gastrodin is the main active ingredient obtained from the Chinese herb,* Tianma* (*Gastrodia elata* Bl.) [8–10]. Gastrodin is considered to have several beneficial properties. Gastrodin has been suggested to be effective as an anticonvulsant and analgesic and is a sedative effective against vertigo, general paralysis, epilepsy, and tetanus [11]. Clinical studies have shown that gastrodin has a good effect on treatment of vertigo and was able to improve hemodynamics, which quickly eliminated dizziness, vertigo, nausea, vomiting, tinnitus, and other symptoms caused by cervical spondylosis and atherosclerosis. Besides, gastrodin may improve microcirculation and cardiovascular compliance and promote fibrinolytic activity and anti-ischemic-reperfusion injury [12]. Additionally, gastrodin could also improve blood pressure, blood rheology, endothelin, and other indicators in patients with hypertension [5]. Some studies have shown that gastrodin inhibited cardiac hypertrophy and fibrosis through inhibiting ERK1/2 signaling pathway and activation of GATA-4 [6]. This study examined the effect of gastrodin on SBP and elucidated its possible mechanism via the activation of PPAR*γ*.

Our investigation showed that treatment with gastrodin for 4 weeks was able to lower the SBP in SHR and did not affect the general health of rats, including body weights, food consumption, and heart rate. The reduction of SBP by gastrodin was associated with the remarkable increase in the level of cardiac PPAR*γ* and the subsequent reduction of the hypertensive effects of ACE and AT1R which can also be locally synthesized in the heart.

Renin-angiotensin-aldosterone system (RAAS) is an important factor in regulating blood pressure, within which there exists a proteolytic cascade. Circulating renin cleaves its substrate angiotensinogen to form the decapeptide angiotensin I (Ang I), which is converted by angiotensin-converting enzyme (ACE) to angiotensin II (Ang II). As the main mediator of RAAS, Ang II acts as a vasoconstrictor and thus elevates blood pressure. It also stimulates the release of aldosterone (ALD) which mediates sodium and water retention by directly acting at the distal tubule and eventually elevating the blood pressure level. The function of Ang II is mediated by the plasma membrane receptor AT1 (AT1R). AT1R stimulates vasoconstriction, vascular cell hypertrophy and hyperplasia, sodium retention, and reactive oxygen species generation. Furthermore, Ang II interacts with AT1R, which results in left ventricular remodeling, alterations in the morphology and mechanical properties of the vasculature, and the development of endothelial dysfunction [1, 13]. Thus, our finding that gastrodin improves blood pressure by targeting and inhibiting Ang II, ALD, ACE, and AT1R does not only have therapeutic significance in clinical management of hypertensive patients; but it may also enhance our understanding on the molecular mechanism underlying the antihypertensive effect of the traditional Chinese medicine.

As aforementioned, ACE, Ang II, AT1R, and ALD are the most important components in RAAS. In our study, we showed that gastrodin could reduce the concentration of Ang II and ALD in serum (Figures 6(a) and 6(b)), decrease the mRNA expression of AT1R (Figure 7(b)), and lower the protein level of AT1R (Figure 6). Surprisingly, gastrodin did not improve the cardiac function in spite of its effect on the expression of the abovementioned proteins (Table 1). It is possible that the intervention time was too short, which only brought changes in the level of protein molecules, and was not enough to cause changes in tissue function.

Peroxisome proliferator-activated receptor (PPAR) *γ* is a nuclear hormone receptor [14]. PPAR*γ* is trans-activated by its agonists that have been reported to be able to lower blood pressure [15–18]. Some clinical studies have shown that the PPAR*γ* agonist telmisartan could inhibit ACE and block AT1R [19, 20]. Besides, PPAR*γ* agonist also blocked the action of Ang II [19, 21]. PPAR*γ* ligands such as rosiglitazone could also reduce the blood pressure in hypertensive rats, increase urinary aldosterone excretion, reduce heart-to-body weight ratio, and diminish aldosterone-induced heart hypertrophy [22]. Therefore, PPAR*γ* not only downregulates the expression of ACE and AT1R, but also blocks the action of Ang II and ultimately inhibits adrenal aldosterone synthesis/secretion.

Therefore our findings suggest that the activation of PPAR*γ* by gastrodin inhibited the expression of ALD and Ang II that led to the reduction in SBP level.

We found that gastrodin was able to increase the mRNA expression of PPAR*γ*, as well as increasing its protein synthesis (Figures 7(a) and 7(b)). Thus, we do not rule out the possibility that gastrodin could reduce blood pressure by activating PPAR*γ* target, since activated PPAR*γ* intervenes with the important components of RAAS, such as ACE, Ang II, AT1R, and ALD. What is more, clinical studies have demonstrated that gastrodin can improve the blood pressure level in patients with hypertension. This study was designed to explore the mechanism of lowering blood pressure. With regard to this, we did not include positive controls in this experiment. Although gastrodin may lower blood pressure by inhibiting RAAS, it can be inferred from our data that the inhibition was not strong enough to improve the overall cardiac function. This can be partially attributed to gastrodin's limited inhibition of RAAS which needs further investigation.

## 5. Conclusion

Despite our incomplete understanding of mechanisms involved in beneficial effects of gastrodin, the results of this study clearly demonstrated that gastrodin injected intraperitoneally at the dose of 100 mg/(kg·d) for 4 weeks decreased the SBP in SHR. Furthermore, this study showed that gastrodin intervened with RAAS effectively, including lowering the levels of Ang II and ALD in serum, reducing the mRNA expression of AT1R in myocardium, and decreasing the protein synthesis of AT1R. Meanwhile, gastrodin also increased the mRNA expression of PPAR*γ* and its protein synthesis. According to previous studies, PPAR*γ* could regulate RAAS and has antihypertensive effects. Therefore, the antihypertensive mechanism of gastrodin may be attributable to the direct intervention of RAAS, or indirect inhibition of RAAS via activation of PPAR*γ*. This needs to be further explored.

## Figures and Tables

**Table 1:** Cardiac function (means ± SEM).

	IVS; d (mm)	IVS; s (mm)	LVID; d (mm)	LVID; s (mm)	LVPW; d (mm)	LVPW; s (mm)	EF (%)
WKY	1.29 ± 0.07	2.21 ± 0.29	6.35 ± 0.43	3.77 ± 0.58	1.64 ± 0.15	2.48 ± 0.16	69.95 ± 8.40
SHR	1.70 ± 0.18	2.33 ± 0.37	6.24 ± 0.80	4.42 ± 1.23	2.02 ± 0.26	2.41 ± 0.47	55.16 ± 17.32
GAS	1.53 ± 0.24	2.09 ± 0.22	6.73 ± 0.42	4.88 ± 0.49	1.87 ± 0.25	2.35 ± 0.21	51.95 ± 7.87

After 4-week treatment, there was no difference in cardiac function among the three groups.
